# Deeper mechanistic insights into epitaxial nickelate electrocatalysts for the oxygen evolution reaction

**DOI:** 10.1039/d3cc00325f

**Published:** 2023-03-02

**Authors:** Ellen M. Kiens, Min-Ju Choi, Luhan Wei, Qiyang Lu, Le Wang, Christoph Baeumer

**Affiliations:** a MESA+ Institute for Nanotechnology, University of Twente, Faculty of Science and Technology P.O. Box 217 7500 AE Enschede The Netherlands c.baeumer@utwente.nl; b Physical and Computational Sciences Directorate, Pacific Northwest National Laboratory Richland WA 99354 USA le.wang@pnnl.gov; c School of Engineering, Westlake University Hangzhou 310030 P. R. China luqiyang@westlake.edu.cn; d Research Center for Industries of the Future, Westlake University Hangzhou 310030 Zhejiang P. R. China; e Peter Gruenberg Institute and JARA-FIT, Forschungszentrum Juelich GmbH 52425 Juelich Germany

## Abstract

Mass production of green hydrogen *via* water electrolysis requires advancements in the performance of electrocatalysts, especially for the oxygen evolution reaction. In this feature article, we highlight how epitaxial nickelates act as model systems to identify atomic-level composition–structure–property–activity relationships, capture dynamic changes under operating conditions, and reveal reaction and failure mechanisms. These insights guide advanced electrocatalyst design with tailored functionality and superior performance. We conclude with an outlook for future developments *via operando* characterization and multilayer electrocatalyst design.

## Abstract 2

Epitaxial oxide thin films enable electrocatalyst design, *e.g.* for the oxygen evolution reaction, based on atomic-level structure–property–functionality relationships.

## Graphical abstract

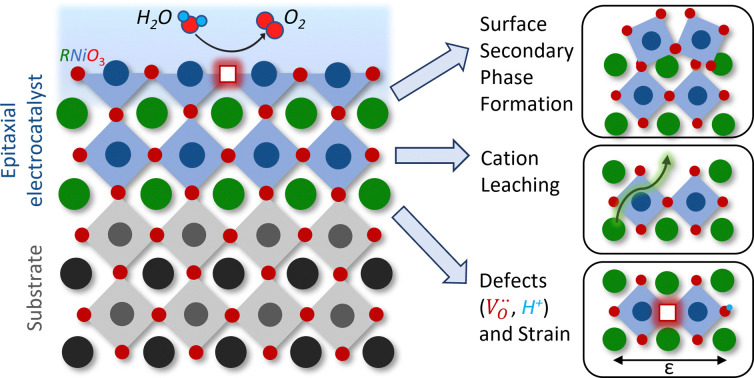

## 1. Introduction

Mass production of green hydrogen through water electrolysis and electrochemical CO_2_ reduction are widely anticipated pathways to reach carbon neutrality. Yet both hydrogen generation and CO_2_ reduction require an oxidation reaction at the anode, which is typically the oxygen evolution reaction (OER). The OER is kinetically complex and often requires a high overpotential, necessitating highly-active electrocatalysts, which must be low cost and also stable under reaction conditions.^1–5^ To date, the understanding of the complicated reaction and failure mechanisms remains incomplete, which impedes the development of electrocatalysts with tailored functionality and superior performance.

An attractive material class for OER electrocatalysts contains the perovskite oxides of the general formula ABO_3_, with rare earth or alkaline earth elements as A-site cations and transition metals (TMs) as B-site cations.^6^ Among perovskite oxides, nickelates (*R*NiO_3_, where *R* represents a rare-earth lanthanide element) have garnered special attention, because of comparably high activity^7^ and suitable earth-abundance. Their excellent performance can be attributed to the following unique characteristics: (1) relatively weaker bond strength between Ni and OH* compared to other first-row TMs,^8^ (2) e_g_ electron filling close to unity,^9^ (3) small or negative charge transfer energy,^10^ and (4) strong hybridization between Ni 3d and O 2p orbitals.^11^ These electronic structure descriptors, to which we will return below, identify perovskite nickelates as one of the most promising class of OER electrocatalysts.

Recent experimental observations have revealed that perovskite oxide electrocatalysts – including nickelates – show a range of dynamic changes rather than staying static during OER.^12–15^ The complicated modifications observed for both surfaces and bulk of nickelate electrocatalysts include the exchange of lattice oxygen and oxygen species between the electrocatalyst and the electrolyte (so-called lattice oxygen mechanisms), the formation and annihilation of ionic defects, which can also be regarded as ion (de-)intercalation processes similar to the electrode materials for aqueous batteries, as well as dynamic surface reconstructions. These dynamic changes during the OER can greatly impact the electrocatalytic activity and stability of nickelate electrocatalysts.

This situation is further complicated by the observation that enhanced catalyst activity seemingly comes at the cost of a decreased stability and lifetime of the catalyst,^15–20^ which implies that good catalyst materials typically degrade very quickly, resulting in a rapid loss of the desired catalytic activity. This inverse correlation between stability and activity of catalyst materials involved in electrochemical processes presents a major obstacle for applications.

Researchers therefore face the challenge of identifying specific factors in the composition, crystal structure, and electronic structure of (nickelate) electrocatalysts that lead to a refined explanation of these dynamic processes, which would enable the design of catalysts with optimized activity *and* stability. Conventionally, electrocatalysts are fabricated as (nano) particles or electrodeposited thin films. These typically have heterogeneous surface composition, ill-defined crystallographic surface phase and exposed facets, which makes extracting intrinsic activity and stability trends difficult. In contrast, epitaxial thin films with well-defined microstructures and surfaces/interfaces offer a valuable platform for elucidating the origins of these dynamic processes.^6,21,22^

In this feature article, we highlight the recent progress in the field on the mechanistic understanding obtained by using epitaxial nickelate OER electrocatalysts. We start by summarizing the current state-of-the-art regarding electrocatalyst activity trends, the role of lattice oxygen participation, and the formation of defects and new “active” phases. We introduce epitaxial thin films as a valuable and informative model platform to identify descriptive structure–property–functionality relationships. We further discuss the remaining unresolved questions and challenges confronting the utilization of the epitaxial nickelate system. We end this review by projecting future directions that enable deeper insights into the dynamic changes of OER electrocatalysts through *operando* characterization, and ultimate tuning of material properties and functionality using multilayer approaches.

## 2. The quest for mechanistic understanding of dynamic changes during OER

**Fig. 1:**
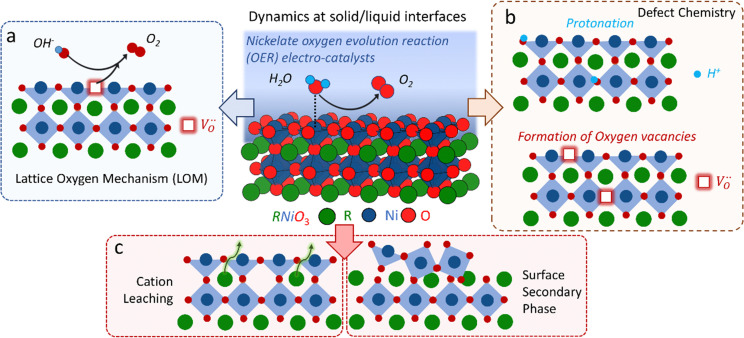
Dynamic changes of nickelate OER electrocatalysts with the perovskite crystal structure. (a) Lattice oxygen mechanism; (b) formation of ionic point defects; (c) surface compositional change and formation of secondary phases.

The classical empirical description of electrocatalytic reactions including the OER assumes a reaction at a solid electrode/liquid electrolyte interface with static composition and structure of the solid. Only little attention has been paid to the “internal life” of the electrocatalysts, *i.e.*, the composition and electronic structure of the surface and subsurface, which may change as a function of potential and time, especially for TM oxide (TMO) based electrocatalysts. There has been mounting evidence that shows the surface and the “bulk” (at least several nanometers from the interface into the electrocatalysts) can participate in the electrochemical reaction, and often determines the kinetics and performance of these electrocatalysts.^15,23–27^ Therefore, a holistic picture considering the solid/liquid interfaces, as well as the constituent ions and ionic defects of TMOs, becomes important for understanding the reaction mechanism and guiding the predictive design of nickelate OER electrocatalysts. Due to the participation of electrocatalyst “bulk” during the electrochemical reactions, a wide range of dynamic mechanisms and phenomena have been observed. These dynamic behaviors of nickelate electrocatalysts indicate that at the OER condition (*e.g.*, *E* ≥ 1.55 V *vs.* RHE (Reversible Hydrogen Electrode)), both the surface and the near-surface region of these electrocatalysts can be very different from the “pre-catalyst” (the parent perovskite oxide before any electrochemical treatment) in both chemical composition and crystal structure. During extended operation, the compositional or structural dynamic changes may further evolve. These dynamic changes, potentially driven by the reorganization of cationic and anionic defects, will drastically alter the electronic structure and active sites of the electrocatalysts. In this section, we will first briefly summarize bulk electronic descriptors of the OER activity that have been shown to be relevant for perovskite oxides. Next, we will review three important aspects regarding the dynamic changes of nickelate electrocatalysts, which are (1) the role of lattice oxygen in OER mechanisms (so-called lattice oxygen mechanism); (2) formation of ionic point defects (oxygen vacancies 
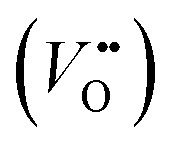
/protons (H^+^)); (3) formation of secondary phases at the near-surface region. Schematics of these three dynamic behaviors are summarized in Fig. 1. To conclude the section, we describe why the use of epitaxial thin films as model systems can help answer unresolved questions in the field.

### 2.1 Bulk descriptors of the OER

As the OER activity and reaction kinetics depend on the electronic structure of the perovskite oxide electrocatalysts, several approximate descriptors relating bulk electronic structure and catalytic activity have been identified, such as the orbital filling of the frontier orbitals and relative orbital positions.^9,10,28,29^ For example, the bonding strength of intermediates is determined by the filling of the antibonding orbital formed between the oxygen species of the adsorbates and TM.^29^ The formation and filling of this antibonding orbital are highly dependent on the e_g_ orbital filling of the TM cations. Hence, the activity of perovskite oxides towards the OER was proposed to follow a volcano-like dependence on the e_g_ orbital filling, with the optimum close to unity, following the Sabatier principle.^9,29^ Not only the occupancy of the e_g_ orbitals influences the binding of intermediates, their chemisorption is also determined by the geometry of the occupied orbitals. The d_3*z*^2^−*r*^2^_ orbital points towards the adsorbates leading to more spatial overlap with the adsorbate O 2p orbitals compared to the d_*x*^2^−*y*^2^_ orbitals.^30^ Hence, a preferred occupancy of these orbitals, *e.g.* through orbital polarization lifting the degeneracy of the e_g_ energy states, will strongly influence the chemisorption and the OER activity, as will be discussed later. In addition to the occupancy and degeneracy of the e_g_ bands, their relative positions also influence the chemisorption and OER activity. For example, suppressing Jahn–Teller distortions in Mn-containing catalysts with spinel structure lifts up the occupied degenerated e_g_ orbitals to higher energy, thereby leading to stronger overlap with the O 2p adsorbate orbitals when compared to Jahn–Teller distorted catalysts, which enhances OER activity.^31,32^ The relative positions of the TM 3d and O 2p have been proposed as a descriptor for OER activities as well. TMOs with perovskite structure typically are materials with mixed ionic and covalent character with a large degree of TM–oxygen orbital mixing (often referred to as TM–oxygen hybridization). The charge transfer energy, defined as the energy difference between the O 2p non-bonding and the TM 3d orbitals, is a measure of the covalency and believed to correlate with the OER activity in a linear manner. Low charge transfer energy, corresponding to high covalency, favors the OER activity.^10,28^ Other descriptors for the OER activity are closely related to defects in the catalyst bulk, such as distortion and 
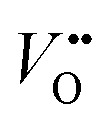
.^12,33^

### 2.2 The role of lattice oxygen in OER mechanisms

Conventionally, the OER in alkaline electrolyte is believed to occur by the so-called adsorbate evolution mechanism (AEM), which means that the reaction follows a consecutive reaction pathway *via* OH* → O* → OOH* → O_2_(g) (* denotes adsorbed species) with TM cations as the main adsorption and reaction sites. In the AEM picture, the lattice oxygen anions of the perovskite oxides are regarded as inert building blocks that do not directly participate in the reaction. In 2016, several groups introduced the lattice oxygen mechanism (LOM) based on theoretical and experimental work.^23,34,35^ It differs from the AEM by the consideration that the O_2_ molecule reaction product can form by taking one oxygen from the lattice and leaving an 
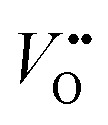
 as shown in Fig. 1a. Interestingly, the authors used LaNiO_3_ nickelate as a model system for their first computational study.^35^ They showed that for LaNiO_3_, the reaction barrier (theoretical overpotential) was much lower for the LOM compared to the AEM. In a later contribution, the same group showed that taking the LOM into account will drastically alter the volcano plot of activity *vs.* B-site 3d TM in LaBO_3_ (B = V, Cr, Mn, Co, Ni and Cu).^36^ Considering the LOM puts LaNiO_3_ at the top of the volcano plot of ternary perovskite oxides. Although the LOM was explained by using LaNiO_3_ as a computational model system, the first experimental demonstration of this new mechanism was on perovskite cobaltates (*e.g.*, La_1−*x*_Sr_*x*_CoO_3−*δ*_).^23,34^ Grimaud *et al.*^23^ directly showed the evolution of O_2_ species involving lattice oxygen by using *in situ*^18^O isotope labelling mass spectrometry, and Mefford *et al.*^34^ showed that the activity scales with the density of 
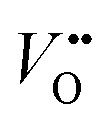
. Presumably, following the computational volcano plot we should see a more important role of lattice oxygen for nickelates compared with cobaltates. However, direct probing on the fraction of AEM/LOM in the total reaction and a comparison between perovskites with different B-site cations is still lacking. Furthermore, for the LOM, it remains yet to be clarified whether only the surface lattice oxygen anions can evolve to oxygen gas molecules, or whether there is a “critical thickness” where all oxygen anions are active within a certain layer. Another important aspect of the LOM is the formation of 
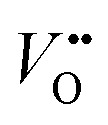
, which is one of the intermediate steps of the OER. Therefore, the ease of 
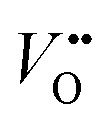
 formation is of great importance for the activation of the LOM. This highlights the role of ionic point defects in the reaction pathways and activity of nickelates. However, the understanding of the defect chemistry of nickelates under the OER condition, *e.g.*, a quantitative correlation between the 
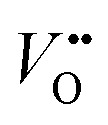
 concentration (ideally with depth resolution) and the applied potential, is still incomplete.

### 2.3 Formation of ionic point defects

Experimental evidence shows that nickelates always contain non-negligible amounts of 
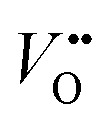
, and that oxygen exchange can occur under sufficient electrochemical driving forces.^37^ Compared to 
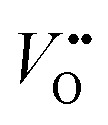
, H^+^ incorporation (protonation) can occur more readily in nickelates in aqueous solution since the hydration process is spontaneous and can compensate the total energy cost for protonation reaction. Recent work by Tang *et al.*^38^ shows experimental evidence that 
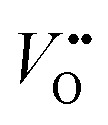
 and H^+^ can co-exist in a perovskite thin film electrocatalyst in alkaline electrolyte. Furthermore, extensive studies show that protonation can be triggered either chemically or electrochemically in nickelate thin films, and that the incorporation of H^+^ can completely change the electrical conductivity (from electronic/metallic to ionic/insulating) and lattice constant of nickelates.^38^ Therefore, the role of point defects as schematically shown in Fig. 1b should be considered when examining the electrocatalytic activity. However, there are several open questions on the room-temperature defect chemical equilibrium and dynamics. For instance, the equivalent of a “Brouwer diagram”, *i.e.*, a quantitative relationship between defect concentration and applied electrochemical driving force (or the activity of oxygen) is still lacking for *R*NiO_3_ in contact with a liquid electrolyte system. This gap of understanding greatly impedes the inclusion of a “defect concentration” term in the modeling of electrochemical reaction kinetics, which has already been considered and widely adopted for treating high-temperature electrochemical reaction kinetics. Furthermore, a detailed study of the defect concentration profile from the solid/liquid interface to the bulk is still lacking. A quantitative understanding of the depth of the near-surface region that can form either 
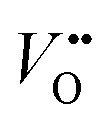
 or H^+^ (or both), and its dependence on the electrochemical driving force and chemical environment, will be important for implementing “defect engineering” in nickelate electrocatalysts.

### 2.4 Formation of secondary phases at the near-surface region

It is well accepted that the near-surface region of TMO-based electrocatalysts can undergo substantial changes in chemistry and phase under sufficiently large electrochemical driving forces. The detailed mechanism or the degree of this dynamic change has been shown to be dependent on the composition and electronic structure of the “pre-catalyst”, as indicated in Fig. 1c. For instance, it has been reported that the cation leaching process, to which we will return later, can be directly correlated with the electronic structure descriptors.^39^ Effectively, for most TMO-based electrocatalysts, the surface becomes a secondary phase under OER conditions, which must be treated differently from the bulk. For nickelates, the surface secondary phase formation might be very subtle. The identification of the dynamic changes thus requires characterizations with high spatial and chemical resolution, which has been demonstrated by our own work (Baeumer *et al.*^14^) and will be discussed in detail later in this review. Although the surface morphology change might be subtle, the altered properties, especially the electronic structure, of the surface secondary phase will directly determine the OER overpotential. Nevertheless, the open question here is how to predict the formation of secondary phases based on the key properties (or descriptors) of the “pre-catalyst”, which might include both the bulk properties (electronic structure, chemical composition, *etc.*) and the surface properties (surface termination, exposed crystal facet, *etc.*).

### 2.5 The need for epitaxial thin films as model system for studying dynamic changes

In order to resolve the dynamic changes of nickelate electrocatalysts during the OER, we propose that epitaxial thin films with well-controlled geometry, composition, surface termination and strain state are a valuable platform. Such films can be constructed with up to atomic precision using deposition techniques such as pulsed laser deposition (PLD) and oxide molecular beam epitaxy (MBE) with *in situ* growth monitoring as described in more detail elsewhere.^22,40–43^ The versatility and control provided by these techniques allows for fabrication of epitaxial thin films as a clean model system without the use of carbon additives or binders, and enables fabrication of materials distinguished by the absence of grain boundaries and complex morphologies. Moreover, control of surface termination and exposed crystallographic facet allows for direct comparison to density functional theory (DFT) calculations. The full control of nickelate samples from the bulk to the surface is of vital importance to develop understanding of intricate connections between multiple phenomena. Furthermore, extracting relevant information from advanced characterization tools can be easier on model epitaxial thin films compared to powder samples, as discussed in Section 4.1. The advantages of using nickelate epitaxial thin films will be showcased by focusing on our own work as examples in this review.

## 3. Epitaxial thin films as a well-controlled and informative platform for elucidating structure–property–function relationships and factors governing the dynamic changes during OER

Below, we discuss recent progress on structure–property–function relationships in epitaxial perovskite nickelate electrocatalysts for the OER. First, we discuss how epitaxial strain can be used to fine-tune crystal field splitting and electronic–structure OER descriptors like the covalency. Then, we turn to modifying the surface composition selectively with atomic layer control to assess the influence of the surface termination for the transformation toward an OER-active surface phase. We finish the chapter with a discussion of compositional variation on the perovskite A- and B-site, which help optimize the electrocatalytic performance and reveal fundamental composition-activity trends.

### 3.1 Epitaxial strain to fine-tune electronic structure and orbital filling

**Fig. 2:**
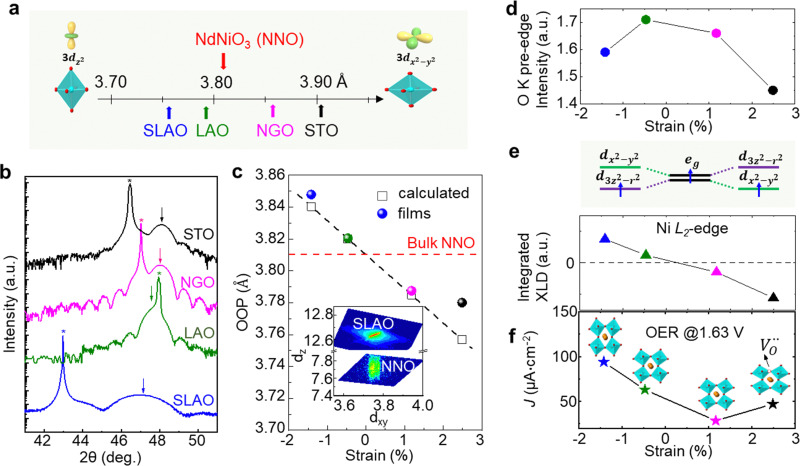
(a) Pseudo-cubic lattice parameters for bulk NdNiO_3_ and single crystalline substrates, including SrLaAlO_4_ (SLAO), LaAlO_3_ (LAO), NdGaO_3_ (NGO), and SrTiO_3_ (STO). The inset shows a schematic diagram of octahedral distortion induced by bi-axial compressive and tensile strain. (b) XRD *θ*−2*θ* scans around the (002) diffraction peak of 5 nm NNO films deposited on different substrates. Film and substrate peaks are noted by arrows and stars, respectively. (c) Experimentally obtained out-of-plane lattice parameter values (colored circles) of NdNiO_3_ films as a function of strain. Strained out-of-plane lattice parameters of NdNiO_3_ films estimated from the Young's modulus are presented by black open squares. The inset displays a reciprocal space map of NdNiO_3_ on SLAO. (d) O K pre-edge intensity of NdNiO_3_ films as a function of strain. (e) Schematic electronic configuration of e_g_ orbital and integrated XLD as a function of strain. (f) OER current density at 1.63 V *vs.* RHE of NdNiO_3_ films under different biaxial strains. The inset in (f) shows schematic illustrations of octahedral distortions induced by strain. Reprinted (adapted) with permission from ref. 41. Copyright 2019, American Chemical Society.

Strain can be introduced by depositing pseudomorphic epitaxial films on single-crystal substrates with different lattice constants. The effect of strain on the OER activity of NdNiO_3_ was studied in our previous work (Wang *et al.*^44^). We used PLD to deposit 5 nm thick NdNiO_3_ thin films on four single-crystal substrates: SrLaAlO_4_, LaAlO_3_, NdGaO_3_, and SrTiO_3_. For these substrates, the in-plane misfit strain of NdNiO_3_ films varies from −1.39% (compressive) to +2.49% (tensile) depending on the substrate, as deduced from the pseudocubic lattice constants of bulk NdNiO_3_ and the four substrates (Fig. 2a). A typical reciprocal space map shown in the inset of Fig. 2c reveals a coherent growth of NdNiO_3_ films on these substrates. Fig. 2b shows the X-ray diffraction (XRD) *θ*−2*θ* scans around (002) peaks of NdNiO_3_ films and the respective substrates. The peak shift as a function of substrate lattice parameter indicates that the substrate-induced in-plane misfit strain is accommodated by the elastic deformation (out-of-plane expansion or contraction) of the NdNiO_3_ films. Fig. 2c summarizes the experimentally obtained and calculated out-of-plane lattice constants of NdNiO_3_ films as a function of strain. It can be seen that the calculated out-of-plane lattice constant of NdNiO_3_ matches well with the experimental value for the film grown on LaAlO_3_, *i.e.* the substrate inducing the minimum in-plane strain. This indicates a very low concentration of point defects such as 
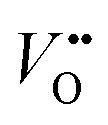
 exist in NdNiO_3_ on LaAlO_3_. On the other hand, the experimental out-of-plane lattice constants of NdNiO_3_ films grown on the remaining substrates are larger than the calculated values which suggests the formation of point defects due to the larger lattice mismatch. Besides showing the largest difference between the experimental and calculated out-of-plane lattice constants, NdNiO_3_ on SrTiO_3_ exhibits the highest resistivity at room temperature and the lowest intensity of the O K pre-edge observed in X-ray absorption spectroscopy, as shown in Fig. 2d. These results suggest the highest 
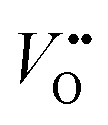
 concentration is present in NdNiO_3_ on SrTiO_3_ (Fig. 2d). The orbital occupancy of strained NdNiO_3_ films was probed by X-ray linear dichroism (XLD = *E*_ab_ − *E*_c_), a technique that uses differential absorption of horizontally and vertically linear polarized light to examine electronic properties. Fig. 2e shows the XLD results of NdNiO_3_ films as a function of strain. For the NdNiO_3_ films under compressive strain, a positive XLD value was obtained, indicating preferential d_3*z*^2^−*r*^2^_ orbital occupancy. The negative integrated XLD intensity for NdNiO_3_ films under tensile strain indicates the preferred occupancy of the d_*x*^2^−*y*^2^_ orbital, resulting in stronger Ni–O chemisorption and lower OER activity (Fig. 2f). This is the reason why the NdNiO_3_ films under compressive strain show higher OER activity than the NdNiO_3_ films under tensile strain. Bak *et al.*^33^ probed the effect of octahedral distortions on the OER activity of perovskite nickelates, which also depend on the strain. They found that strong distortion of oxygen octahedra results in a considerable increase in electronic states near the Fermi level (*E*_F_), making the charge transfer between TM and oxygen much easier, thereby enhancing OER activity.^33^ This is different for Mn-containing catalysts with spinel structure, in which the OER activity increases with the suppression of the Jahn–Teller distortion.^31,32^ On the other hand, when a tensile strain large enough to generate 
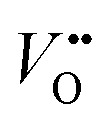
 is applied, the Ni valence can be reduced from Ni^3+^ to Ni^2+^. This leads to e_g_ filling slightly larger than one, which can contribute to the enhanced OER activity observed in NdNiO_3_ on SrTiO_3_. Similar experiments were also performed on other TMOs, demonstrating that strain-induced structural distortion and oxygen deficiencies modify the OER activity of epitaxial TMO films.^42,45^

### 3.2 Tuning surface termination towards active surface phase formation

**Fig. 3:**
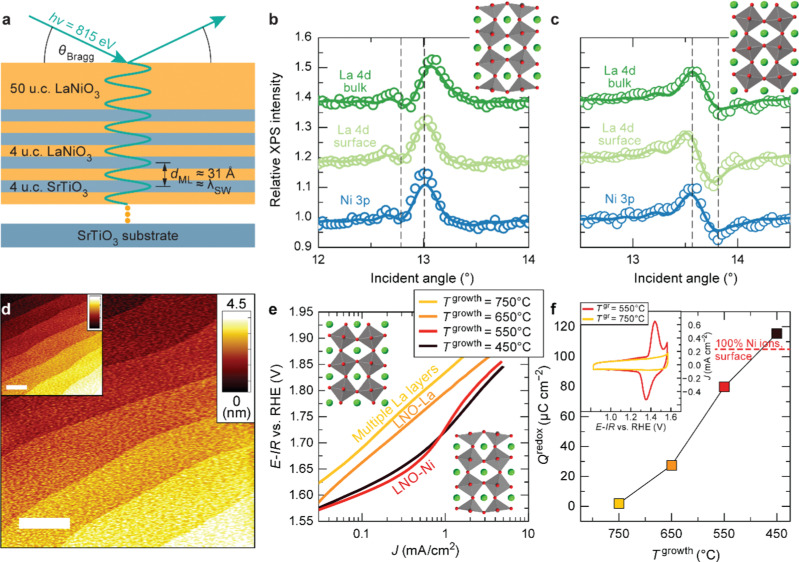
(a) Schematic diagram of LaNiO_3_ thin films on a superlattice of LaNiO_3_ (4 unit cells (u.c.)) and SrTiO_3_ (4 u.c.). SW-XPS rocking curves of La 4d and Ni 3p for LaNiO_3_ top layers grown at (b) 450 and (c) 650 °C. Atomic force microscopy images for (d) as-deposited LaNiO_3_ film (20 nm) grown at 550 °C and (inset) after cyclic voltammetry (CV). (e) Tafel-like plot for LaNiO_3_ films with different surface termination. (f) Redox charge for conversion from Ni^2+^ to Ni^3+^ during CV. (inset) CV curves in the Ni^2+^–Ni^3+^ redox area for LaNiO_3_ films deposited at 550 and 750 °C. Reproduced with permission from ref. 10.

As discussed in Part 2, nickelate OER electrocatalysts often go through dynamic changes of the structure and chemical composition, especially at the electrochemical (solid/liquid) interface and the near-surface region of the oxide. While conventional particulate electrocatalysts (even nanoparticles) can be difficult to control precisely, our recent work (Baeumer *et al.*^14^) shows that epitaxial nickelate thin films, with the capability of controlling the surface termination and composition with atomic precision, can be a powerful platform to obtain detailed understanding on the dynamic changes during the OER. We employed standing-wave X-ray photoemission spectroscopy (SW-XPS), which is an emerging technique capable of depth-profiling the chemical environment in the near-surface region with 1–2 Å depth resolution, to reveal details of the surface chemical composition of LaNiO_3_. Epitaxial layers are an ideal system for this spectroscopic technique because of the opportunity to create smooth films supported superlattices functioning as X-ray mirrors (Fig. 3a). SW-XPS analysis of LaNiO_3_ prepared in different growth conditions revealed predominant NiO_2_ and LaO termination in Fig. 3b and c, respectively. Electrochemical characterization of the same samples revealed that NiO_2_-terminated LaNiO_3_ thin films show much higher OER activity compared to LaNiO_3_ samples with LaO-termination (Fig. 3e). Importantly, the surface morphology of samples before and after determining the OER activity was very similar, both showing atomically smooth terraces (Fig. 3d). Furthermore, redox peaks in cyclic voltammetry tests were only observed in the NiO_2_-terminated LaNiO_3_. Their peak area (which corresponds to the charge transferred across the solid/liquid interface) increases with Ni coverage of the first surface layer of LaNiO_3_. Combining experimental evidence from SW-XPS and electrochemical measurement showed that the different activity can be directly related to the different composition of the surface termination layer. With the insights from both experimental evidence and computational support, the dynamic changes of LaNiO_3_ surfaces were clarified. The difference in surface composition evolves to different surface secondary phases, *i.e.*, NiO_*x*_H_*y*_ (oxy-hydroxide like) and La(OH)_3_ at sufficiently high potential, with the former much more active than the latter. Therefore, this work proves that a very subtle difference in chemical composition (only the first atomic surface layer) could lead to a very large difference in activity, which can be more readily captured by using well-controlled epitaxial thin films as model systems.

### 3.3 Optimizing electrocatalyst functionality *via* cation substitution

**Fig. 4:**
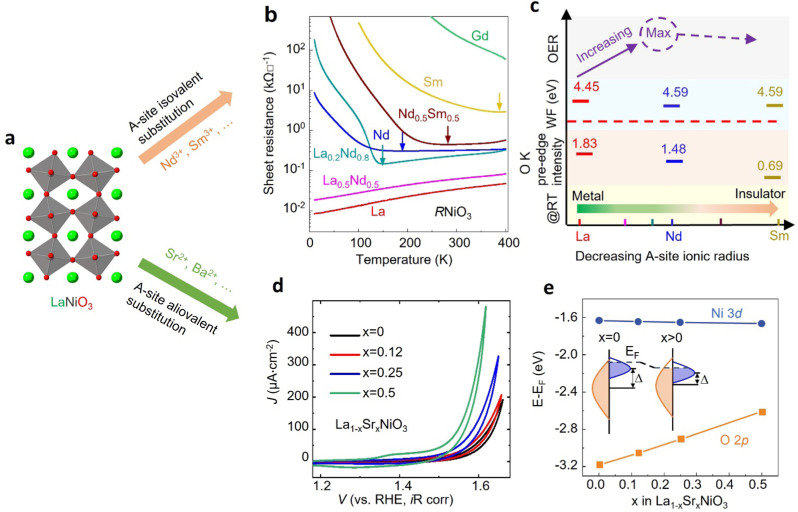
(a) Schematic illustration of LaNiO_3_ crystal structure. (b) Sheet resistance as a function of temperature for perovskite nickelate thin films with different A-site cations grown on SrTiO_3_ substrate. (c) The trends in the OER activity, work function, O K pre-edge intensity, and room temperature conductivity with decreasing the ionic radius of A-site cations in nickelates. Reproduced with permission from ref. 43. Copyright 2018, Wiley-VCH. (d) CV curves of La_1−*x*_Sr_*x*_NiO_3_ thin films with *x* = 0, 0.12, 0.25, and 0.5. (e) Calculated average onsite energies of Ni 3d and O 2p orbitals with *E*_F_ as a reference. The inset shows a schematic energy band diagram of LaNiO_3_ (*x* = 0) and La_1−*x*_Sr_*x*_NiO_3_ (*x* > 0). *Δ* denotes the charge transfer energy. Reproduced with permission from ref. 44. Copyright 2019, Wiley-VCH.

Cation substitution is a common strategy to tune the electronic structure of nickelates for optimizing OER activity. Taking LaNiO_3_ (Fig. 4a) as an example, the cation substitution can be classified based on the doping sites, namely A-site, B-site, or dual sites. Although A-site cations in nickelates are not considered as active sites for OER, they can indirectly contribute to the OER by altering lattice distortion, 
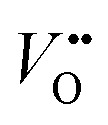
 concentration, Ni valence state, and the Ni 3d and O 2p hybridization. According to the doping elements, the A-site substitution can be divided to isovalent or aliovalent substitution. In 2018, we (Wang *et al.*^46^) used PLD to synthesize a series of RNiO_3_ (where R = La, La_0.5_Nd_0.5_, La_0.2_Nd_0.8_, Nd, Nd_0.5_Sm_0.5_, Sm, and Gd) thin films on SrTiO_3_ substrates and investigated the effect of A-site isovalent substitution on OER activity. We found that decreasing the ionic radius of A-site elements in RNiO_3_ leads to a decrease in the electronic conductivity, which is consistent with a decrease in Ni–O bond covalency due to bending of the Ni–O–Ni angle. Moreover, reducing the ionic radius of A-site elements induces the formation of 
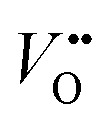
 (the decrease of the O K pre-edge intensity), which increases the average occupancy of the antibonding e_g_ orbital and lowers *E*_F_. This is favorable for the OER activity (Fig. 4c). One year later, we (Liu, Wang *et al.*^47^) used MBE to deposit a set of La_1−*x*_Sr_*x*_NiO_3_ (*x* = 0, 0.12, 0.25, 0.5) thin films on LaAlO_3_ substrates and investigated the effect of A-site aliovalent substitution on the OER activity. Cyclic voltammetry curves of La_1−*x*_Sr_*x*_NiO_3_ thin films in Fig. 4d show a clear trend towards improved OER activity as *x* increases, with *x* = 0.5 showing the most active OER activity. DFT calculations (Fig. 4e) clearly demonstrate that Sr doping in LaNiO_3_ induces an upward energy shift of O 2p band relative to *E*_F_, strengthening Ni 3d–O 2p hybridization and decreasing the charge transfer energy. Both contribute to enhancing the OER activity.

**Fig. 5:**
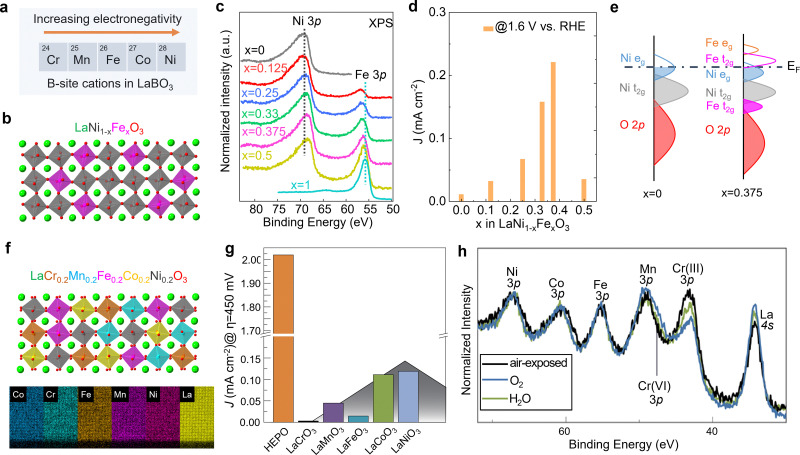
(a) Five common first-row transition metals used as B-site cations in LaBO_3_ and the trend of electronegativity. (b) Schematic illustration of LaNi_1−*x*_Fe_*x*_O_3_ crystal structure. (c) *In situ* Ni 3p and Fe 3p core level XPS spectra of LaNi_1−*x*_Fe_*x*_O_3_ films with *x* = 0, 0.125, 0.25, 0.33, 0.375, and 0.5. (d) OER current density of LaNi_1−*x*_Fe_*x*_O_3_ films measured at 1.6 V *vs.* RHE. (e) Schematic energy band diagram with corresponding electronic structure near *E*_F_. Reprinted (adapted) with permission from ref. 32. Copyright 2021 American Chemical Society. (f) Schematic illustration of LaCr_0.2_Mn_0.2_Fe_0.2_Co_0.2_Ni_0.2_O_3_ and energy-dispersive X-ray spectroscopy maps of a LaCr_0.2_Mn_0.2_Fe_0.2_Co_0.2_Ni_0.2_O_3_ high entropy perovskite oxide (HEPO) thin film. Scale bar: 5 nm. (g) OER current density of HEPO and five parent perovskites thin films measured at 1.68 V *vs.* RHE. (h) TM 3p core level ambient pressure XPS spectra of the HEPO thin film in two different atmospheres. (f–h) Reprinted (adapted) with permission from ref. 49.

Unlike A-site cations, the B-site cation, *i.e.*, the Ni site of *R*NiO_3_ nickelates, is believed to directly participate in the electrochemical reaction, either as active site for OER or through the covalent interaction with the active oxygen site. Recently, we (Wang *et al.*^48^) further explored the effect of B-site substitution, namely partially replacing Ni with other transition metals, on the OER activity. The electronegativity of the first-row transition metals increases when moving from left to right across the periodic table. Thus, Ni is the most electronegative element compared to other four cations Cr, Mn, Fe, and Co (Fig. 5a). Partially replacing Ni with Fe (Fig. 5b), or Cr, Mn, Co is expected to reduce the Ni valence state, which was verified in our work (Wang *et al.*^48^). A whole set of epitaxial LaNi_1−*x*_Fe_*x*_O_3_ (*x* = 0, 0.12, 0.25, 0.33, 0.375, 0.5, 1) solid solutions were synthesized by MBE. Chemical compositions and electronic structures were directly characterized by *in situ* XPS without air exposure. Fig. 5c clearly shows that, with doping Fe in LaNiO_3_, charge transfer from Fe to Ni occurs in these solid solutions, reducing Ni from Ni^3+^ to Ni^2+^ and oxidizing Fe from Fe^3+^ to Fe^4+^. DFT calculations indicate the high-valent Fe^4+^ states can significantly contribute to the density of states around E_F_ (Fig. 5e), increasing the total TM 3d bandwidth *via* d-orbital coupling in Ni–O–Fe bridges. This change in electronic structure decreases the energetic cost to accept/donate electrons at the adsorbate–electrocatalyst interface, boosting the OER activity (Fig. 5d). Moreover, the Ni^2+^/Ni^3+^ gradually increases with *x* in LaNi_1−*x*_Fe_*x*_O_3_, which reduces the Ni 3d bandwidth and finally results in the bandgap opening. The decrease of total TM 3d–O 2p orbital hybridization induces a quick drop in OER activity at *x* = 0.5. Thereby, a volcano-like OER catalytic trend was observed with 37.5% Fe substitution in LaNi_1−*x*_Fe_*x*_O_3_ as the most active composition.

The potential beneficial effect of substitutional doping can be maximized using multiple elements on the same lattice site by using a solid–solution approach. Such multi-compositional perovskite oxides are frequently referred to as high-entropy perovskite oxides due to the increased configurational entropy induced by the mixing of several elements on the same crystallographic lattice site. These oxides may benefit from compositionally-fine-tuned electronic and atomic structure and the synergistic effects of multiple cations simultaneously/consecutively participating in electrocatalytic reactions. Furthermore, the high entropy may lead to phase stabilization according to the free energy of formation, resulting in increased stability against dissolution.^50^ We recently found that (001)-oriented LaCr_0.2_Mn_0.2_Fe_0.2_Co_0.2_Ni_0.2_O_3−*δ*_ epitaxial thin films outperformed the OER activity of all parent perovskites with single B-site cation by a factor of 16 to 670 (Fig. 5g).^49^ We speculated that this enhancement of electrocatalytic activity may be the result of the synergistic behavior of multiple cations, since the adsorption and binding of water molecules to the surface has been shown to induce an appreciable change in oxidation state of Cr, Co, and Ni simultaneously (Fig. 5h). The electronic structure descriptors discussed previously cannot explain the observed activity trend. Therefore, more experimental and theoretical work are needed to capture the complex composition–property–function relationships in this multi-dimensional space.^50^

## 4. Outlook

### 4.1 *Operando* characterization for deeper mechanistic insights

Solid–solid, solid–gas and especially solid–liquid interfaces continuously evolve as a function of applied potentials, ionic concentrations, and time. For electrocatalysts, this implies that true active phases and reaction sites do not “pre-exist” in as-prepared “pre-catalyst” surfaces, but evolve under reaction conditions.^51^ This will be discussed below in more detail for the case of NiO_2_-terminated LaNiO_3_ from the example in Section 3.2. To achieve further understanding of the structure–property–function relationships governing electrocatalyst materials discovery, characterization of chemical composition, atomic arrangement and electronic structure of the interface under operating conditions is therefore necessary.

The term “*operando*” refers to measuring material properties under working conditions, *i.e.*, while the reaction of interest occurs, with simultaneous measurement of reaction rate.^52,53^*Operando* characterization goes well beyond *in situ* or *ex situ* characterization of the as-prepared or even the operated surfaces because the catalyst changes reversibly or irreversibly as a function of potential. Therefore, only *operando* measurements can shed light on the surface properties actually giving rise to electrocatalytic activity. In this section, we will provide an overview of *operando* characterization techniques recently employed for epitaxial thin film electrocatalysts and techniques that exhibit high potential for impactful future use. We include a discussion why epitaxial thin-film samples are particularly useful to extract the relevant information.

**Fig. 6:**
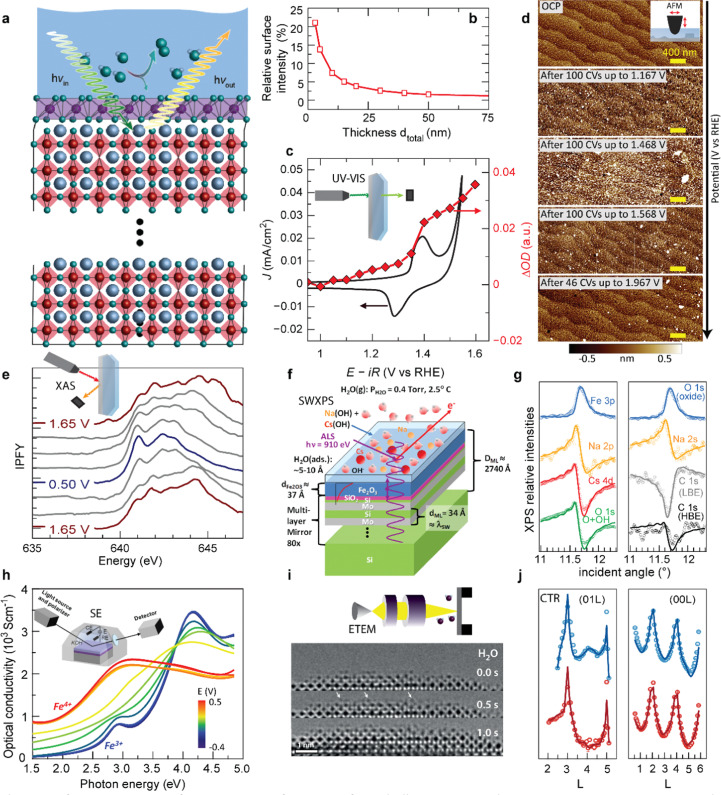
(a) Schematic for extracting surface-sensitive information from bulk-sensitive techniques. A representative perovskite oxide electrocatalyst (red corner-sharing octahedra) exposed to an electrolyte develops a structurally and electronically different surface layer when a potential is applied to drive the oxygen evolution reaction (OER), in this example involving transformation of the top unit cell (violet edge-sharing octahedra), inspired by (ref. 10 and 49). (b) The relative intensity of a 0.8 nm surface layer measured by a bulk-sensitive technique like XAS or UV-Vis spectroscopy increases with decreasing total thickness of the electrode. Reprinted from C. Baeumer, *J. Appl. Phys.*, 2021, **129**, 170901, with the permission of AIP Publishing (c) cyclic voltammetry in the pre-OER region for Ni-terminated LaNiO_3_ (LNO–Ni), along optical density change at *λ* = 500 nm during potential holds. Reproduced with permission from ref. 10. (d) Surface roughening during cycling up to increasingly higher potentials in and 0.5 M H_2_SO_4_ (scale bars are 400 nm). Used with permission of Royal Society of Chemistry, from “Probing the Stability of SrIrO_3_ During Active Water Electrolysis *via Operando* Atomic Force Microscopy”, A. R. Akbashev *et al.*, *Energy Environ. Sci.*, 2023, **14**. (e) Electrocatalyst changes with applied potential tracked by Mn L-edge XAS of electrodeposited MnO_*x*_ films in 0.1 M KOH cycled from 1.65 V to 0.50 V and back to 1.65 V *vs.* RHE. Reproduced (adapted) with permission from ref. 36. Copyright 2017, American Chemical Society. (f) Sample geometry used for SW-XPS analysis. (g) Comparison of experimental rocking curves for various XPS core-level intensities with calculations based on the optimized sample configuration, which allows extraction of sub-nanometer-resolution depth profiles of atomic concentrations. (f and g) Reproduced with permission from ref. 55. Copyright 2014, Springer Nature. (h) Optical conductivity spectra of a La_0.5_Sr_0.5_FeO_3−*δ*_ thin film, recorded at various potentials. The inset shows a schematic representation of the chamber used for the measurements. Reprinted (adapted) with permission from Y. Q. Tang *et al.*, *ACS Appl. Mater. Interfaces*, 2022, **14**, 18486–18497. Copyright 2022 American Chemical Society. (i) ETEM investigation of the La_0.6_Sr_0.4_CoO_3−*δ*_/H_2_O interface, showing a representative images of a time sequence in 0.5 Pa of H_2_O. Highly mobile adatoms are highlighted by white arrows. Reproduced with permission from ref. 9. (j) (01L) and (00L) crystal truncation rods measured at 0.5 V (blue) and 1.5 V *vs.* RHE (red). Open points and solid lines denote the experimentally measured intensities and the best-fit results from the fitting process, respectively. Used with permission of Royal Society of Chemistry, from “Towards identifying the active sites on RuO_2_ (110) in catalyzing oxygen evolution”, R. R. Rao *et al.*, *Energy Environ. Sci.*, 2017, **10**, 2626–2637.

Typical surface transformation giving rise to the electrocatalytic activity are confined to the surface,^14^ yet many *operando* characterization techniques are bulk-sensitive, *i.e.*, averaging the information from a thin surface layer with the underlying volume of the electrocatalyst. This hampers sensitivity to small structural or chemical changes in the surface layer. Fig. 6a and b shows a guiding principle to extract surface-sensitive information from a nominally bulk-sensitive technique, described in more detail in ref. 14 and 54. The relative intensity of a thin surface layer depends on the total thickness of the electrode. Using epitaxial thin films with unit-cell thickness control, one can extract the surface signal from a series of *operando* experiments with various sample thicknesses. We employed this technique successfully using bulk-sensitive UV-Vis spectroscopy in transmission geometry (Fig. 6c). The optical density of the electrocatalyst at a characteristic photon wavelength changes reversibly at the previously discussed Ni redox potential. Comparison to reference samples and systematic thickness-dependent measurements revealed a phase transition of a unit-cell-thin layer to a Ni oxyhydroxide-like layer.^14^ This transformation towards an edge-sharing octahedral arrangement of the Ni atoms in a sub-nanometer surface layer is key for the high electrocatalytic activity observed for NiO_2_-termination because it does not occur for LaO-termination.

A similar approach to tune bulk-sensitive methods towards surface-sensitivity may be applied to X-ray absorption spectroscopy (XAS). XAS allows extraction of oxidation states, coordination geometry and number, and elemental composition from peak shapes, positions, intensities, and spin–orbit splitting.^56^ In the past, *operando* XAS was already employed, for example, to track valence changes at OER-relevant voltages of a layered manganese oxide film prepared by electrodeposition (Fig. 6e).^55^ X-ray absorption spectra revealed reversible yet hysteretic Mn redox. Oxidation to a mixed Mn^3+/4+^ valence preceded the oxygen evolution, and additional analysis of the O spectra showed changes in Mn–O hybridization with different applied potentials, where an e_g_ occupancy near one likely corresponds to the highest hybridization. In the future, *operando* XAS may therefore aid in identifying mechanistic OER activity trends based on the electronic structure of epitaxial nickelates under operating conditions. Yet, for epitaxial thin films, designing appropriate experimental cells is more challenging compared to conventional electrocatalyst materials because fabrication on X-ray-transparent membranes or a cell design with very thin liquid compartments are necessary to minimize attenuation of incoming and outgoing X-ray, as discussed in more detail elsewhere.^51^

In addition to studying activity trends, characterization of degradation pathways becomes increasingly important for selecting ideal electrocatalyst materials since an inverse activity–stability relationship seems to exist as introduced previously. Akbashev *et al.*^57^ recently investigated the degradation of SrIrO_3_, a highly active electrocatalyst, during the oxygen evolution reaction (OER) using *operando* electrochemical atomic force microscopy (EC-AFM). This material serves as a model system for degradation studies of perovskite ABO_3_ oxides, exhibiting both A-site cation leaching and transition metal (B-site) dissolution. EC-AFM can track surface morphology changes and dissolution, and epitaxial thin films are particularly suited for mechanistic studies because they provide a smooth, step-terraced surface morphology with unit-cell precise thickness control of the as-prepared state. For the epitaxial SrIrO_3_, the thickness remained identical during operation in H_2_SO_4_ up to 2 V *vs.* RHE, implying that no dissolution occurred in these conditions and in contrast to severe dissolution in alkaline media.^58^ In H_2_SO_4_, particulates appear at ∼1.17 V *vs.* RHE and disappear at 1.5–1.6 V *vs.* RHE, as shown in Fig. 6d. These particulates affect the surface roughness but the step-terraced morphology remains. The authors proposed that these particulates are SrSO_4_, and discuss the formation and removal of the particulates in the context of Sr leaching observed by XPS and mass spectroscopy. Importantly, they also conclude that the stability of perovskite oxide electrocatalysts under operating conditions might be improved by suppressing A-site leaching a common degradation mechanism to which we will return below. For iridium based electrocatalysts, dissolution of the A-site element compared to Ir is highly dependent on their structure.^59^ Electrocatalysts composed of strong edge- and face-sharing octahedra are more robust against A-site dissolution.^60^ Furthermore, balancing A- and B-site dissolution by B-site substitution can help to maintain crystal structure and prevent catalyst degradation.^61^*Operando* characterization may aid to identify these degradation mechanisms and related material properties to ultimately design more efficient and robust catalysts.

As previously discussed, the electrocatalytic performance is not only controlled by the surface but also by the defect chemistry of the underlying layer, which also changes during operation due to parasitic (de-)intercalation reactions. Therefore, *operando* probes for the type and concentration of point defects such as 
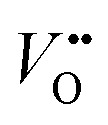
 and protons are needed. Tang *et al.*^38^ recently developed an *operando* spectroscopic ellipsometry approach, allowing the tracking of the concentration of holes as a function of the intercalation potential (Fig. 6h). They characterized epitaxial La_0.5_Sr_0.5_FeO_3−*δ*_ thin films and indirectly quantified the concentration of electron holes for different electrochemical potentials using the Fe oxidation state. Combining the experimental results with a defect chemistry model, they extracted estimates for the concentration of H^+^ and 
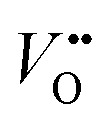
 in the epitaxial layer as a function of potential. This approach may help to tune electrocatalyst properties through property-control by ionic intercalation, and can reveal the changes in electronic structure during electrochemical preconditioning.

We now turn towards future pathways for *operando* characterization that may lead to even deeper analysis or higher sensitivity and interface-specificity. As discussed in the previous section, SW-XPS is capable of depth-profiling the chemical environment in the near-surface region with 1–2 Å depth resolution. Nemšák *et al.*^62^ reported a strategy to extend SW-XPS to solid–liquid interfaces using an ∼1 nm thick hydrated layer on α-Fe_2_O_3_, combining ambient-pressure XPS and SW-XPS. They found that SW-XPS at the solid–liquid interface allows sub-nm resolution of the spatial arrangement of chemical species, in this case the position of Cs and Na cations in the hydrated layer, as well as local potential energy variations along the direction perpendicular to the interface (Fig. 6f and g). Later, the same research group followed up with a pioneering *operando* study, revealing that SW-XPS is even possible in the presence of an electrolyte meniscus with ∼20 nm thickness while applying electrochemical potentials.^63^ In this example, they demonstrated the oxidation and hydroxylation of an 8 nm thick Ni film while maintaining sub-nanometer depth resolution using the increasingly popular dip-and-pull geometry, which was introduced in the seminal paper by Axnanda, Crumlin *et al.*^64^ The same depth resolution can be now achieved using near total reflection geometry, which is especially attractive because it provides all the strengths of a standard SW-XPS without the need of an artificial multilayer mirror, making the technique more broadly applicable.^65^

For atomic-level spatial resolution, environmental transmission electron microscopy (ETEM) is a key development as it enables the direct imaging of surface processes on the atomic scale under exposure to ambient gases. When operated in H_2_O conditions, a thin water layer condensates on the perovskite surface, as pioneered in the group of Jooss.^66^ The primary electron beam of the TEM causes anodic polarization of the perovskite catalyst, resulting in close to relevant OER potentials. Weber *et al.*^13^ and Lole *et al.*^66^ recently used this technique to reveal the dynamics at the La_0.6_Sr_0.4_CoO_3−*δ*_/H_2_O and the La_0.6_Sr_0.4_MnO_3−*δ*_/H_2_O interfaces, where the TM ions are found to be highly mobile under OER potentials; a snapshot of the time sequence for this interface is shown in Fig. 6i.^13^ Such dynamics present an important future contribution for our understanding of electrocatalytic activity at continuously transforming electrocatalyst surfaces.

Lastly, we suspect that surface XRD and the investigation of crystal truncation rods (CTR) will lead to new fundamental insights when employed for epitaxial thin film electrocatalysts. CTR analysis was already successfully employed to study the surface structure and binding of reaction intermediates at single crystal surfaces, as showcased for the RuO_2_ surface in Fig. 6j, where the diffraction data was fitted with a surface model including the presence of an *OO intermediate on specific Ru sites.^67,68^ Such analyses, especially when combined with *ab initio* modelling and microkinetic modelling, may therefore lead to a more complete picture of the reaction intermediates preceding the rate determining step and potential-dependent surface structures and coverages, all of which are urgently needed for enhanced mechanistic understanding of OER electrocatalysts.

### 4.2 Layer-by-layer control of functionality and performance

To further enhance the performance of nickelates and perovskite electrocatalysts in general, both the activity and stability must be considered because of the typically-observed inverse activity–stability relationship. As stated before, epitaxial thin films are a valuable platform to develop structure–property–function relationships that might provide design rules to optimize both activity and stability simultaneously. It was recently shown that this inverse activity–stability relationship is not universal and can be overcome by careful inspection of facet-dependent activity and stability. For example, for LaNiO_3_, the (111) facet is both more active and more stable than other facets, a trend that could be revealed studying epitaxial thin films synthesized on differently oriented substrates.^69^ Epitaxial thin films also enable the fabrication of material combinations with hybrid properties by interfacing different perovskite oxides with unit cell thickness control.^22^ Such heterostructures offer even more degrees of freedom to engineer stable and active electrocatalysts that overcome the inverse stability–activity relationship.

**Fig. 7:**
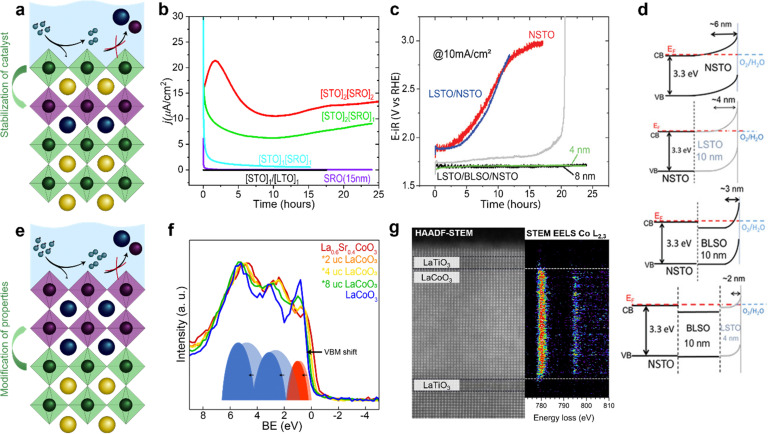
Epitaxial multilayers offer opportunities to engineer both active and stable OER catalysts using various methods. (a) Hybrid structures can prevent catalyst dissolution. (b) Subsurface layers of SrRuO_3_ (SRO) can activate SrTiO_3_ (STO). Subscripts indicate the number of unit cells of STO (SRO) in the surface (subsurface). The current density of SrTiO_3_ with an inactive subsurface layer of LaTiO_3_ (LTO) and of a thin film of SrRuO_3_ over time are shown for comparison. Used with permission of Royal Society of Chemistry, from “Activation of ultrathin SrTiO_3_ with subsurface SrRuO_3_ for the oxygen evolution reaction”, A. R. Akbashev *et al.*, *Energy Environ. Sci.*, 2018, **11**, 1762–1769 (ref. 54. (c and d) Band engineering using layers of La doped BaSnO_3_ (BLSO) and La-doped SrTiO_3_ (LSTO) facilitates charge transport and enhances the lifetime of epitaxial catalysts grown on Nb-doped SrTiO_3_ (NSTO). Reproduced with permission from ref. 63. Copyright 2019, Wiley-VCH. (e) Interfacial engineering allows for modification of surface properties without changing the chemical composition of the catalyst. (f) Interfacial engineering tunes the Co–O covalency of hybrid bilayers of 2–8 unit cells (u.c.) LaCoO_3_ on 20 nm La_0.6_Sr_0.4_CoO_3_ as shown using X-ray photoelectron of the valence band. Reproduced with permission from ref. 65. (g) Interfacial engineering results in a change in Co oxidation state near an epitaxial interface as observed with high angle annular dark field (HAADF) scanning transmission electron microscopy (STEM) with electron energy loss spectroscopy (EELS). Reprinted figure with permission from Araizi-Kanoutas *et al.*, *Phys. Rev. Mater.*, 2020, **4**, 026001. Copyright 2020 by the American Physical Society (ref. 68).

As previously discussed, the electrochemical properties of epitaxial thin films are influenced by the surface layer as well as the subsurface layer. Engineering of the subsurface layer allows for optimization of the activity and stability. For example, electronic transport in thin film or heterostructure electrodes can be manipulated by engineering the interfacial depletion layers and corresponding potential barriers. Baniecki *et al.*^70^ showed that buried epitaxial layers can be exploited to engineer the depletion layer and enhance the overall performance of epitaxial catalysts. Heterostructures of La-doped BaSnO_3_ and La-doped SrTiO_3_ on Nb-doped SrTiO_3_ substrates were used as support of few-unit-cell thin La_0.7_Sr_0.3_CoO_3−*δ*_ catalysts layers. The introduction of La-doped BaSnO_3_ and La-doped SrTiO_3_ underneath the electrochemically active La_0.7_Sr_0.3_CoO_3−*δ*_ results in a narrower depletion layer and facilitates p-type character in the catalyst that greatly enhances its activity. This results in fast transport kinetics, even accompanied by enhanced stability as shown in Fig. 7c and d. Furthermore, engineering of the band edge has also been found to greatly enhance the performance of photoanodes in photoelectrochemical water-splitting. For example, the introduction of subsurface dipoles by placement of 1 unit cell LaAlO_3_ in SrTiO_3_ leads to improved efficiency.^71^

In addition to the manipulation of band bending at the solid/liquid interface, electronic properties of the active surface can be tuned in epitaxial multilayers as schematically shown in Fig. 7e. In our work (Heymann *et al.*^72^), we showed that the enhanced Co–O covalency at the surface of hybrid epitaxial bilayers of La_0.6_Sr_0.4_CoO_3_ and LaCoO_3_ influences its activity. We evaluated valence band spectra of hybrid bilayers of 2–8 unit cells LaCoO_3_ on top of 20 nm La_0.6_Sr_0.4_CoO_3_ and their parent compounds, as shown in Fig. 7f. The valence band of La_0.6_Sr_0.4_CoO_3_ shows more overlap between the O 2p (blue) and Co 3d (red) contribution compared to LaCoO_3_, which indicates higher Co–O covalency for La_0.6_Sr_0.4_CoO_3_. The spectra obtained for the hybrid bilayers also show large overlap between O 2p and Co 3d bands (more than expected based on a linear combination of the parent spectra) indicating interfacial hybridization in the bilayer catalysts. As Mott–Schottky analysis indicates hole accumulation for all investigated bilayers and both parent compounds in the OER regime, the observed activity of the fabricated bilayers is attributed to the increased Co–O covalency obtained by interfacial hybridization, rather than band bending at the solid/liquid interface.

Similar interfacial hybridization effects are exploited by Akbashev *et al.*^58^ to activate OER-inactive SrTiO_3_ by burying highly active but unstable ultra-thin SrRuO_3_ layers in the subsurface. The placement of such an active subsurface layer in a stable host perovskite is shown schematically in Fig. 7a. The introduction of a SrRuO_3_ layer in SrTiO_3_ as a host material is expected to result in Ru 4d states that hybridize with SrTiO_3_ electronic states and lie within the band gap of SrTiO_3_, as predicted by DFT calculations. The authors show that as little as one unit cell of SrRuO_3_ in the subsurface greatly enhances the activity of the catalyst compared to SrTiO_3_, as indicated in Fig. 7b. Furthermore, they show that the lifetime of the multilayered catalyst is enhanced compared to bare SrRuO_3_ because the dissolution of SrRuO_3_ is suppressed by the SrTiO_3_ surface layer. The stability of the multilayer greatly increases as the SrTiO_3_ capping layer thickness is increased from 1 to 2 unit cells, while its activity decreases with increasing capping layer thickness. Hence, a clear trade-off between activity and stability seems to exist in these hybrid multilayered catalysts. Other promising material combinations to achieve an enhancement of activity by subsurface doping have been identified computationally by Zhang *et al.*^73^

A similar method of enhancing activity by the introduction of a subsurface layer is applicable to epitaxial catalysts for the oxygen reduction reaction. Eom *et al.*^74^ showed that single unit cell thick layers of SrMnO_3_ in LaMnO_3_ can greatly enhance its activity. Changing the A-site cation from La to Sr affects the electronic configuration of Mn and therefore the activity of the catalyst. The authors found that a layer of SrMnO_3_ in the subsurface shows a greater enhancement in activity compared to placement of this layer at the surface. The lower activity of the catalyst when Sr is present in the surface layer is attributed to SrO formation upon exposure of the catalyst to oxygen, as observed by ambient pressure X-ray photoelectron spectroscopy. This work serves as a demonstration of how the introduction of different sub-surface layers may activate a catalyst without changing the chemical composition at the surface and therefore compromising its stability.

In addition to the stabilization of active catalysts by the prevention of dissolution with capping layers, or activation of inactive catalysts by placement of more active subsurface layers, as shown in Fig. 7a, interfacial engineering offers even more opportunities to tune the properties of stable catalysts to enhance activity, as shown in Fig. 7e. For example, the oxidation state of B-site atoms in epitaxial perovskites changes near the interface of perovskites with a different B-site atom, as shown in Fig. 7g.^75^ This offers opportunities to manipulate the oxidation state, structural distortions, orbital filling and covalency of ultrathin catalytic layers without changing their chemical composition. As the chemical composition remains unchanged, this is a promising strategy to overcome the observed inverse activity–stability relationship by activating chemically stable surfaces through remote doping, which may be extended from these Co- and Ru-based examples to epitaxial nickelate layers. Due to the control of the growth of epitaxial multilayers, these films serve as promising model systems to provide guidelines for the design of catalytic core–shell nanoparticles that show superior stability and activity.

In conclusion, epitaxial nickelate layers have emerged as an ideal model to study intrinsic structure–property–functionality relationships, which will be exploited by translating materials design criteria towards high-surface-area synthesis pathways. It was found that engineering the strain state, the crystallographic facet and the surface composition can greatly affect the catalytic activity. Further optimization can be achieved through substitutional control of the composition and resulting electronic structure. The next steps forward will likely be achieved by designing future generation catalysts with the insights from *operando* characterization of the active state of the surface and subsurface (defect) structure and composition, and by interfacing multiple electrocatalysts in a core–shell structure to enhance functionality without changing the surface chemical composition.

## Conflicts of interest

There are no conflicts to declare.

## Supplementary Material
